# Selection of *Beauveria bassiana* (Hypocreales: Cordycipitaceae) strains to control *Xyleborus affinis* (Curculionidae: Scolytinae) females

**DOI:** 10.7717/peerj.9472

**Published:** 2020-07-03

**Authors:** Jesús E. Castrejón-Antonio, Patricia Tamez-Guerra, Roberto Montesinos-Matías, Maria J. Ek-Ramos, Paul M. Garza-López, Hugo C. Arredondo-Bernal

**Affiliations:** 1 Universidad Autónoma de Nuevo León, Facultad de Ciencias Biológicas, San Nicolás de los Garza, Nuevo León, México; 2 SENASICA, Centro Nacional de Referencia de Control Biológico, Tecomán, Colima, México; 3 Instituto de Ciencias Agropecuarias, Universidad Autónoma del Estado de Hidalgo, Tulancingo, Hidalgo, México

**Keywords:** Ambrosia beetles, Conidial germination, Cuticle degrading enzymes, Growth rate, Hydrophobicity, Principal components analysis

## Abstract

**Background:**

*Xyleborus affinis* Eichhoff (Coleoptera: Curculionidae) is an ambrosia beetle reported to affect avocado trees (*Persea americana* Mill.). The use of the entomopathogenic fungus (EPF) *Beauveria bassiana* (Bals.-Criv.) Vuill. for ambrosia beetle control represents an alternative to insecticides.

**Methods:**

This study was designed in two stages to select *B. bassiana* strains with potential to control *X. affinis* females. In the first stage, 19 *B. bassiana* Mexican strains from EPF collection, isolated from Coleoptera (CHE-CNRCB, http://www.gob.mx/senasica/documentos/coleccion-de-hongos-entomopatogenos), were tested. Analyses included radial growth rate, conidial yield, spore germination, and germ tube length. Results were analysed by Principal Component Analysis (PCA) to identify clusters within favourable growth phenotypes. For the second stage, 10 selected strains were re-analysed for virulence-related metabolic characteristic, including cell wall-bound cuticle-degrading enzymes–Pr1-like proteases and β-N-acetyl glucosaminidases (NAGase) chitinases, conidial hydrophobicity and monopolar germination parameters. A second PCA analysis was run for those virulence parameters analysed, and upon results strains CHE-CNRCB 44, 171, 431 and 485 were selected and tested against *X. affinis* females. Females were treated with a 1 × 10^8^ conidia mL^−1^ suspension (recommended rate), using a Potter Tower.

**Results:**

All strains showed insecticidal activity, inducing up to 58% mortality; about 30% dead beetles developed aerial mycelia (CHE-CNRCB 485) and the fastest mortality rate was *t_0_* = 1.95 (CHE-CNRCB 44).

**Conclusion:**

Since all selected strains showed virulence against *X. affinis* females, results indicated the possibility of selecting *B. bassiana* strains based on multiple metabolic attributes, as a preliminary test to perform bioassays against order-related target insects.

## Introduction

Globally distributed ambrosia beetles represent trees-associated aggressive pests of forest ecosystems. Indeed, several native species are a serious threat to agriculture (Popa et al., 2012; Smith & Hulcr, 2015). In this regard, *Xyleborus* species are considered dangerous pests to North America (Haack & Rabaglia, 2013). In the United States, *Xyleborus glabratus* Eichhoff (Coleoptera: Curculionidae) transmits a vascular disease called *laurel wilt disease* to redbay (*Persea borbonia* L.), which is caused by the fungus *Raffaelea lauricola* T. C. Harr., Fraedrich and Aghayeva (Fraedrich et al., 2008; Harrington, Fraedrich & Aghayeva, 2008). In Mexico, *X. glabratus* and *R. lauricola* are not present, but species such as *X. affinis* Eichhoff (Coleoptera: Curculionidae), which also carries *Raffaelea lauricola* T.C. Harr., Fraedrich & Aghayeva (Ophiostomatales: Ophiostomataceae), is one of the most widespread ambrosia beetles worldwide. It has been reported in the state of Colima, Mexico infesting avocado trees (*Persea americana* Mill.) and in mango orchards (*Mangifera indica* L.) among trees with regressive wilt symptoms (Sobel, Lucky & Hulcr, 2015; Castrejón-Antonio et al., 2017b, 2018).

Although containment strategies for ambrosia beetles control include the use of insecticides (Jones & Paine, 2018), overseas commercialisation of Mexican avocados (Hass variety) restricts pesticide application to this crop (https://www.federalregister.gov/documents/2016/05/27/2016-12586/mexican-hass-avocado-import-program, accessed by 27 November 2019). In fact, legislation in the USA and Europe has led to use-restrictive or use-elimination for many pesticides (Carrillo, Crane & Peña, 2013; Jones et al., 2017; Berger & Laurent, 2019). Biological control is an alternative strategy against ambrosia beetle (Hughes et al., 2015; Dunlap et al., 2017; SENASICA, 2018). Entomopathogenic fungi (EPF) have been used against several borer beetles (Kreutz, Zimmermann & Vaupel, 2004; Wegensteiner, Wermelinger & Herrmann, 2015; Avery et al., 2018). Studies have shown that EPF were infective to ambrosia beetles, including *Xyleborus volvulus* Fabricius (Coleoptera: Curculionidae), *X. bispinatus* Eichhoff (Coleoptera: Curculionidae), *Xylosandrus germanus* Brandford (Coleoptera: Curculionidae), *Xy. crassiusculus* Motschulsky (Coleoptera: Curculionidae) (Castrillo, Griggs & Vandenberg, 2013; Avery et al., 2018; Tuncer et al., 2019).

*Beauveria bassiana* (Bals.-Criv.) Vuill. (Hypocreales: Clavicipitaceae), is a fungus species isolated from borer beetles (Selvasundaram & Muraleedharan, 2000; Wegensteiner, Wermelinger & Herrmann, 2015; Montesinos-Matías et al., 2019a). Popa et al. (2012) confirmed that *B. bassiana* was reported infecting 12 borer beetle species. They discussed that *B. bassiana* infection among ambrosia bark beetles may be related to a better environmental adaptation. Under laboratory condition tests, Carrillo et al. (2015) reported the highest mortality and mycosis percentage against the red bay ambrosia beetle, *X. glabratus* caused by *B. bassiana*–based commercial product Botanigard^®^ES, as compared with other fungal products tested.

Selection of EPF candidate strains for biocontrol purposes is critical for the development of a successful commercial product. It is possible to select fungal strains based on their virulence and survival under unfavourable environmental conditions, relevant to their use as commercial biopesticide (Ravensberg, 2011). Unfavourable abiotic factors including temperature, pH, solar radiation, and relative humidity (RH) may determine success or failure of fungal biopesticide in the field, since they may reduce or limit the fungal propagules viability (Pérez-González et al., 2014; Zhu, Ying & Feng, 2016; Shin et al., 2017). Moreover, factors related to virulence against insect’ pests include germination and infection rate, high sporulation, conidium size, toxin and/or insect cuticle-degrading enzymes production, appressorium formation, and propagules hydrophobicity (Altre, Vandenberg & Cantone, 1999; Montesinos-Matías et al., 2011a, 2011b; Zhang et al., 2011). These factors can be used to select EPF strains constituting parameters of their biocontrol potential against some target insects (Mascarin et al., 2013). Such a practice would be especially valuable when insufficient numbers of insects are available to perform biocontrol bioassays, although not all growth factors will relate to a specific strain virulence. In this regard, Pletamul & Prasertsan (2012) reported that the most virulent *B. bassiana* BNBCRC strain against *Spodoptera litura* L. larvae showed the highest germination rate as well, without considering conidia yield and mycelium radial growth.

In the present study, selection of *B. bassiana* strains was performed by evaluating growth profile (first stage), pathogenic characteristics (second stage), and virulence against *X. affinis* beetle females.

## Materials and Methods

### *Beauveria bassiana* source

Nineteen coleoptera-isolated *B. bassiana* strains were selected from the catalogue from the EPF collection (http://www.gob.mx/senasica/documentos/coleccion-de-hongos-entomopatogenos, accessed 28 January 2019) from the Centro Nacional de Referencia de Control Biológico (CNRCB), located in Colima state, México. Fifteen of the 19 strains were isolated from beetles, from which 13 were the coffee berry borer *Hypothenemus hampei* Wood & Bright (Curculionidae: Scolytinae).

All strains were stored on silica gel at 5 °C to be further grown on Petri dishes containing Sabouraud Dextrose Agar (SDA), supplemented with 1% yeast extract (SDAY), which is commonly used for entomopathogenic fungi culture and assays (Talaei-Hassanloui et al., 2007), and incubated at 27 ± 2 °C in darkness for 20 d.

### *Xyleborus affinis* colony

To establish *X. affinis* colony under laboratory conditions, adult beetles were collected in Comala, Colima, México (19.45965° N, −103.65603° W), from avocado trees showing such signs of ambrosia beetle infestation as decay and wilt. Permit to collect beetles was granted by the Colima State Plant Health Committee (Comité Estatal de Sanidad Vegetal de Colima) and the landowner, Julio Aguirre González. Beetles were identified by using Wood’s (1982) taxonomic keys (Castrejón-Antonio et al., 2017a). Adults were kept in plastic conical 50 mL tubes and fed on artificial avocado sawdust diet (Cooperband et al., 2016), placing five insects per tube and incubating at 25 ± 2 °C for 4 weeks.

### Selection stage 1: *B. bassiana* strains growth profile

For *B. bassiana* strains selection, before performing bioassays against ambrosia beetles, and since germination and infection rates, high conidium size, and sporulation are factors that may correlate with their virulence, mycelium growth rate (mm day^−1^), conidial production (conidia mm^−2^), conidia germination (%), and germ tube length (mm) were analysed on SDAY culture medium. Each experiment was done in triplicate.

### Growth rate

A 6-mm diameter sterile filter paper disc was placed at the centre of a 90-mm Petri dish containing SDAY medium. Based on previous tests, 5 μL of an 8 $\times$ 10^6^ conidia mL^−1^ suspension was added on each disc. All strains were cultured at the same time and incubated at 27 ± 2 °C. Colony diameter size was daily measured, calculating the average (mm d^−1^) of the two perpendicular diameters. Diameter size data of fungal growth were completed when one of the 19 tested strains grown within 0.5 cm of the Petri dish edge. Data were analysed using linear regression model (SPSS, V21), where the slope value was considered as the growth rate.

### Conidia production

*Beauveria bassiana* strains were cultured as described above and incubated at 27 ± 2 °C for 20 d. A 20-mm-diameter sample mycelial plug was taken from the growing plate, halfway between the colony centre and edge (Montesinos-Matías et al., 2011b). Conidia were released from the resulting culture plug by transferring it into a 50 mL conical tube containing 10 mL Tween 80 (0.05%) and vortexed for 1 min (maximum speed) (Vortex-Genie 2, Scientific Industries, Bohemia, NY, USA). Conidia were counted in a haemocytometer.

### Conidial germination

Ten microliters of an 8 $\times$ 10^6^ conidia mL^−1^ suspension were transferred into a conical microtube containing 990 μL of Tween 80 (0.05%), after which it was vortexed at maximum speed (7) for 1 min, and 10 μL of the resulting suspension were cultured in a 17-mm diameter disk of SDAY medium at 27 ± 2 °C for 18 h. After incubation, the disk was transferred onto a coverslip and a drop of blue lactophenol (20 g of phenol, 20 g of lactic acid and 40 g of glycerol mixed in 20 mL distilled water) was added along with a coverslip. Viable propagules percentage was determined by counting 100 conidia (germinated or not), using an optical microscope with a 40$\times$ objective (Safavi et al., 2007). Conidia were counted as viable if germ tube length was twice the conidia diameter size (Wraight, Inglis & Goettel, 2007).

### Germ tube length

Conidial germination tubes were detected from the slides used in the germination tests (18 h). Then, germ tube length from 30 conidia was measured for each strain. Photographs were taken using an Axio Scope A1 microscope with a 40$\times$ objective and an Axiocam ICc 1 camera (Carl Zeiss de México, S.A. de C.V., Ciudad de México, México) and analysed by the Axiovision Release 4.8.2 software.

### Stage 1 data analysis

Mean values of each variable for the 19 strains were standardised to a *Z*-score. Prior to standardisation, data expressed as percentages were transformed using the arcsine function. Data were analysed using one-way analysis of variance (ANOVA) and Tukey’s test. As well as Principal Component Analysis (PCA), using the Spearman method setting of the XLSTAT statistical package (V.2018 for Windows, Addinsoft, NY, USA). PCA correlations were considered significant when the Bartlett’s sphericity test *p* value was ≤0.05. Variables with a correlation coefficient ≥0.6 were considered relevant.

Following growth variables correlation from PCA analysis, 10 strains were selected for further analysis, because they grouped in at least two of the clusters. Strains that met this criterion were CHE-CNRCB 21, 26, 37, 38, 171, 174 and 485. Strain CHE-CNRCB 431 was selected for possessing the highest germination rate, whereas strains CHE-CNRCB 44 and CHE-CNRCB 117 were chosen based on conidia production.

### Selection stage 2: evaluation of fungal virulence factors

Two cell wall-bound cuticle-degrading enzymes–Pr1-like proteases and β-N-acetyl glucosaminidases (NAGase) chitinase (St. Leger et al., 1991; Safavi et al., 2007) were evaluated for the 10 selected *B. bassiana* strains. In addition, conidial hydrophobicity and monopolar conidial germination percentages were assessed (Talaei-Hassanloui et al., 2007). To stimulate enzyme production, strains were grown on plates in a culture medium containing a 10:1 carbon: nitrogen ratio at 27 ± 2 °C (Safavi et al., 2007). Each experiment was performed in triplicate.

### Pr1 protease activity

To determine Pr1 protease activity, 500 μL of Tris-HCl 0.1 M buffer (pH 7.9) (Sigma–Aldrich, St. Louis, MO, USA), containing one mM of *N*-succinyl-alanine-alanine-2-proline-phenylalanine-*p*-nitroanilide substrate (Sigma–Aldrich, St. Louis, MO, USA), was used following protocols reported by Ayala-Zermeño et al. (2015) and Castrejón-Antonio et al. (2017a). For this, 500 μL of a 3 $\times$ 10^8^ conidia mL^−1^ suspension were mixed in a 1,500 µL conical microtube containing Tween 80 (0.05%). Mixture was incubated at 27 ± 2 °C for 10 min and the enzymatic reaction was stopped with 100 μL of 0.1 M HCl. Tubes were then placed on ice for 5 min to stabilise the resulting chromogen and then centrifuged (Centrifuge 5430 R, Eppendorf, Hamburg, Germany) at 15,295×*g* at 4 °C for 5 min. Supernatant optical densities were measured using a NanoDrop 2000 spectrophotometer (Thermo Scientific, Wilmington, DE, USA) at 405 nm. A mixture of Tris-HCl buffer (0.1M), substrate and Tween 80 (0.05%) solution was used as a blank. Protease activity was reported in nmol of nitroaniline mL^−1^ min^−1^ (Castrejón-Antonio et al., 2017a).

### NAGase chitinase activity

To determine NAGase chitinase activity, 200 μL of citrate-K_2_HPO_4_ 0.2 M buffer (pH 5.6) and 200 μL of 1 mg mL^−1^ of *p*-nitrophenyl *N*-acetyl-β-D-glucosaminide (Sigma–Aldrich, St. Louis, MO, USA), were used according to previous reports by St. Leger et al. (1991) and Castrejón-Antonio et al. (2017a). For this, 200 μL of a 3 $\times$ 10^8^ conidia mL^−1^ suspension were mixed in Tween 80 (0.05%) in a 1.5 mL conical microtube. Mixture was incubated in a shaker (Lab-Companion, BS-11, Korea) at 120 rpm and 37 ± 2 °C for 60 min, and enzymatic reaction was stopped by adding 1 mL of 0.02 M NaOH. Microtubes were then centrifuged for 5 min at 15,295 × g at 4 °C (Thermo Scientific, Hamburg, Germany) and optical densities were determined at 400 nm. A mixture containing citrate-K_2_HPO_4_ buffer, substrate, and Tween 80 (0.05%), was used as a blank. NAGase chitinase activity was reported in nmoles of *p*-nitrophenol mL^−1^ min^−1^.

### Hydrophobicity

Conidial hydrophobicity was performed according to Kim et al. (2010). For this, strains were grown on SDAY slant culture tubes (18 $\times$ 150 mm) at 27 ± 2 °C, and after 15 d, culture surface was scraped using 0.1 M KNO_3_ (pre-filtered through a 0.2 μm filtration membrane (Thermo Scientific, Hamburg, Germany)) to release the conidia. The resulting 15 mL suspension was transferred into a 50 mL conical tube and vortexed for 5 min at highest speed (7, Vortex-Genie 2, Scientific Industries, Bohemia, NY, USA). Suspension was then filtered into a new 50 mL corning tube using sterile double-layer gauze and labelled as a stock solution. The conidial stock solution concentration was determined using a haemocytometer (Marienfeld, Lauda-Königshofen, Germany) and 5 mL of a suspension of 1 $\times$ 10^7^ conidia mL^−1^ was prepared. Then, one mL of *n*-hexadecane (Sigma–Aldrich, St. Louis, MO, USA) and three mL of the conidial suspension were added to 10 $\times$ 100 mm glass test tubes. This mixture was vortexed at high speed (6) for 20 s and allowed to stand for 30 min to allow separation of the aqueous and organic phases. Then, one mL of the aqueous phase was carefully extracted with a long neck glass Pasteur pipette and poured into a new glass tube. Conidia were counted using a haemocytometer. Relative hydrophobicity was calculated using the following formula:

$$H\left( \% \right) = \left( {1 - \displaystyle{C \over {{C_0}}}} \right)100\% \;$$

where *H* represents hydrophobicity, *C*_0_ represents the conidial stock suspension, and *C* represents the conidial concentration in the aqueous phase.

### Percentage of monopolar conidial germination

A 1:1,000 dilution was prepared from each 3 $\times$ 10^8^ conidia mL^−1^ suspension used for the enzyme activity evaluation. From these dilutions, 10 μL were used to inoculate a 2-cm diameter disk of culture medium. After inoculation, each disk was incubated at 27 ± 2 °C for 18 h, then the disk was placed onto a clean slide. This sample was stained by adding 50 µL of blue lactophenol and covered with a coverslip. Sample was observed under a microscope at 40×. The conidial percentage showing only a single germ tube was determined by observing 100 conidia (Talaei-Hassanloui et al., 2007).

### Stage 2 data analysis

Mean values of each parameter were clustered using PCA analysis, as described in “Stage 1 data analysis”. Variables showing the highest correlation such as enzymatic activity and hydrophobicity were clustered. Upon clusters, four strains were selected to test their virulence against *X. affinis* females.

### Biocontrol of *X. affinis* by *B. bassiana*

To determine *B. bassiana* pathogenicity, we selected *X. affinis* females from reared colonies of approximately 10–15 d old, assuring females were not too teneral nor too sclerotised (with a similar sclerotisation degree). Females were externally hygienised in order to remove as much as possible those contaminants attached to the insect, either by remaining diet and by microbiota present in the galleries. A cleaning process reported for ambrosials (Castrillo, Griggs & Vandenberg, 2013; Cruz et al., 2018) was used as follows: (1) Tween 80 (0.05%), (2) 70% ethanol, (3) again Tween 80 (0.05%) and (4) 0.1% sodium hypochlorite, with each cleaning lasting 10 s. Insects were allowed to dry for 20 min on sterilised absorbent paper. Once dried, they were separated in 10 individual groups in 150 $\times$ 600 mm sterile glass Petri dishes.

*Xyleborus affinis* females were treated with each *B. bassiana* strain using a Potter Tower (BS00281, Burkard Scientific, Uxbridge, UK) calibrated at 103.4 kPa pressure, which applied a dose of 2 mL of 1 $\times$ 10^8^ viable conidia mL^−1^. Strains conidial viability was determined as previously described. Experimental set-up was a completely randomised design with two replicates (*n* = 10 for each replicate), repeated five times.

After females were sprayed with the conidial suspension, beetles were individually placed into plastic vials (50 mm high and 25.7 mm in diameter), containing a piece of avocado sawdust artificial diet, same as used for rearing purposes. Vials were incubated in a humidified chamber at 27 ± 2 °C and 70 ± 5% humidity for 8 d, replacing the feeding source every 2 d. Vials were then daily observed to identify and collect dead individuals, and mortality rates were registered. Dead beetles were disinfected using the described external hygienization, by vortexing at low speed (2) in each solution for 10 s. Clean insects were then placed in a humidified chamber and incubated to allow *B. bassiana* sporulation for up to 10 d.

Since mortality results were lower than 60%, the time required to reach 40% mortality (LT_40_) was estimated from a plot of accumulated mortality *versus* time, for all strains. Data were fitted to an exponential decaying function (Rodríguez-Gómez et al., 2009) using the following formula:

$$Y = \left( {100 - S} \right)\; {e^{ - k\; \left( {t - {t_0}} \right)}} + S;\; {\rm If}\; t > {t_0}$$

where Y = 100 if 0 ≤ *t* ≤ *t*_0_, where *Y* is percent survival at time *t*, *k* is specific death rate (d^−1^), *t*_o_ is delay time (d) and *S* is estimated asymptotic survival level (%). This model corresponds to a first order differential equation with the indicated time delay, the aforementioned initial condition and the asymptotic value, where $Y \to S$ as $t \to \infty$.

### Infected beetles’ conidial production

Five beetles covered with aerial conidia (not mycelium) removed from each bioassay replicate, were used. In order to have sample representativeness, insects were randomly selected and transferred into two mL Eppendorf tubes with 1.5 mL of Tween 80 (0.05%), and vortexed (maximum speed) for one min. Tubes were maintained at 4 °C for 24 h. After vortexing the tubes for 1 min, six aliquots of 10 μL were removed from the resulting suspension and placed in a haemocytometer to determine the average of conidial number *per* beetle, *per* tested strain (*n* = 4).

### Bioassays analysis

To correct mortality collected data with the control treatment, Abbott’s transformation was used (Abbott, 1925), which resulted in <5% adjustment. Data distribution was confirmed using Kolmogorov Smirnov test and were analysed by ANOVA and Duncan’s multiple range tests. Values that were expressed as percentages were converted using an arcsine transformation prior to ANOVA (Studebaker, 1985). For analysis statistical software SPSS was used (V.20, 2012, Chicago, IL, USA).

## Results

### Stage 1 analysis: growth profile

**Figure 1:**
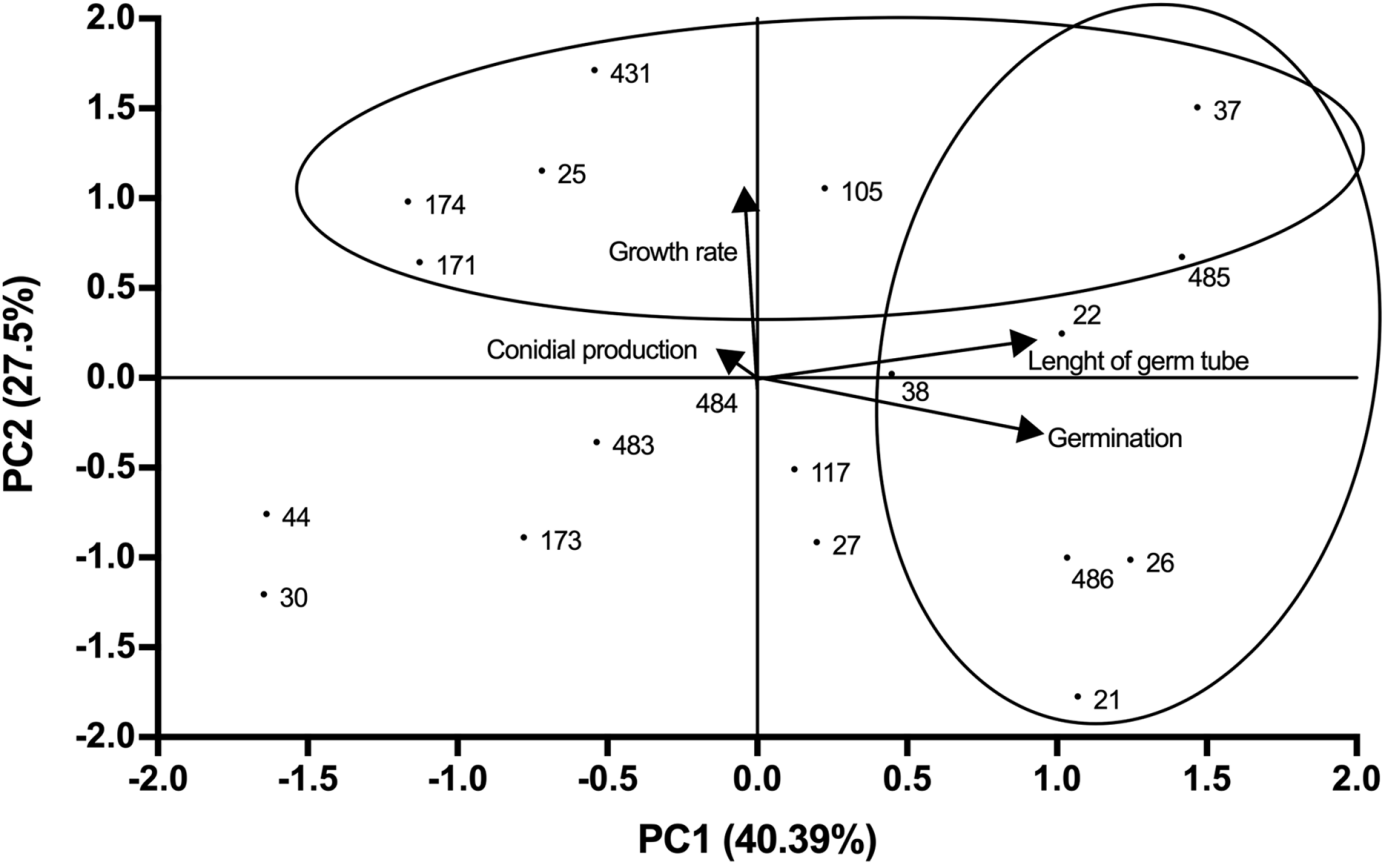
Relationships between the variables, components (PC1 vs. PC2) and distribution of *Beauveria bassiana* strains at the initial selection stage. Each point represents the weighted sum of each strain in its respective component. The arrows indicate the direction and magnitude of the correlation of the variables with the extracted components.

**Figure 2:**
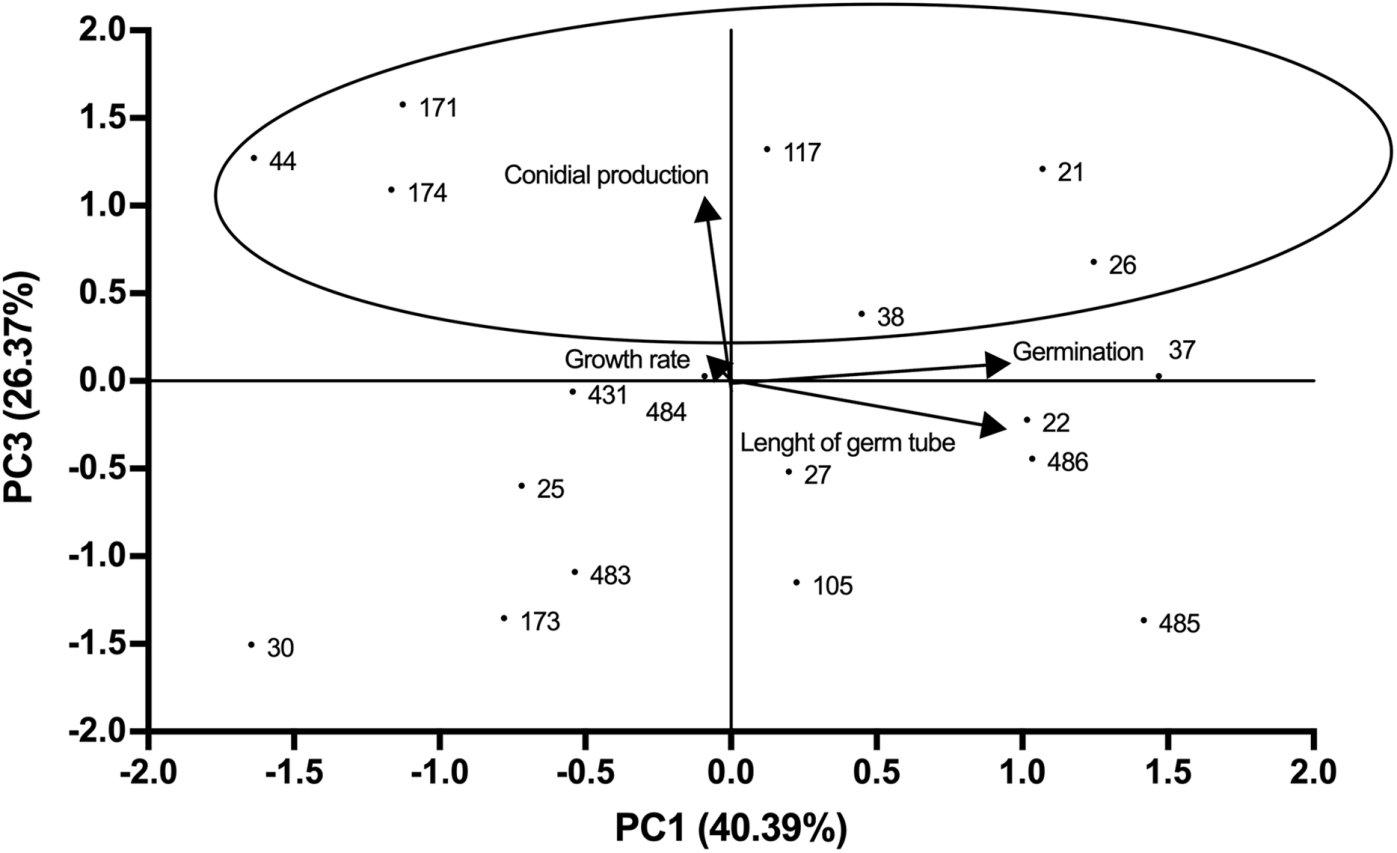
Relationships between the variables, components (PC1 vs. PC3) and distribution of *Beauveria bassiana* strains at the initial selection stage. Each point represents the weighted sum of each strain in its respective component. The arrows indicate the direction and magnitude of the correlation of the variables with the extracted components.

Growth profiles of *B. bassiana* isolates evaluated are shown in supplemental Table S1. PCA indicated grouping of data into three main components that accounted for 94.25% of total variance (Eigenvalue 0.94). The best correlation with component 1 (PC1) was observed for germ tube length and germination (correlation coefficients of 0.89 and 0.91 respectively), which represented most of the total variance (40.39%) (Fig. 1; Table S2). Component 2 (PC2) showed the highest correlation with growth rate (correlation coefficient of 0.98), representing 27.5% of total variance (Fig. 1). Component 3 (PC3) correlated with conidial production, accounting for 26.37% of the total variance (Fig. 2). Based on the weighted sum of each strain for component, those that presented values greater than 0.3 were selected (locked in the ovals). The strains were fitted into three clusters: (1) those characterised by longer germ tubes and higher germination levels (CHE-CNRCB 21, 22, 26, 37, 38, 485 and 486); (2) those with higher growth rates (CHE-CNRCB 25, 37, 105, 171, 174, 431 and 485); and (3) those with higher conidia production (CHE-CNRCB 21, 26, 38, 44, 117, 171 and 174). Strains were grouped based on these results (Table S3).

### Stage 2 analysis: virulence factors

**Figure 3:**
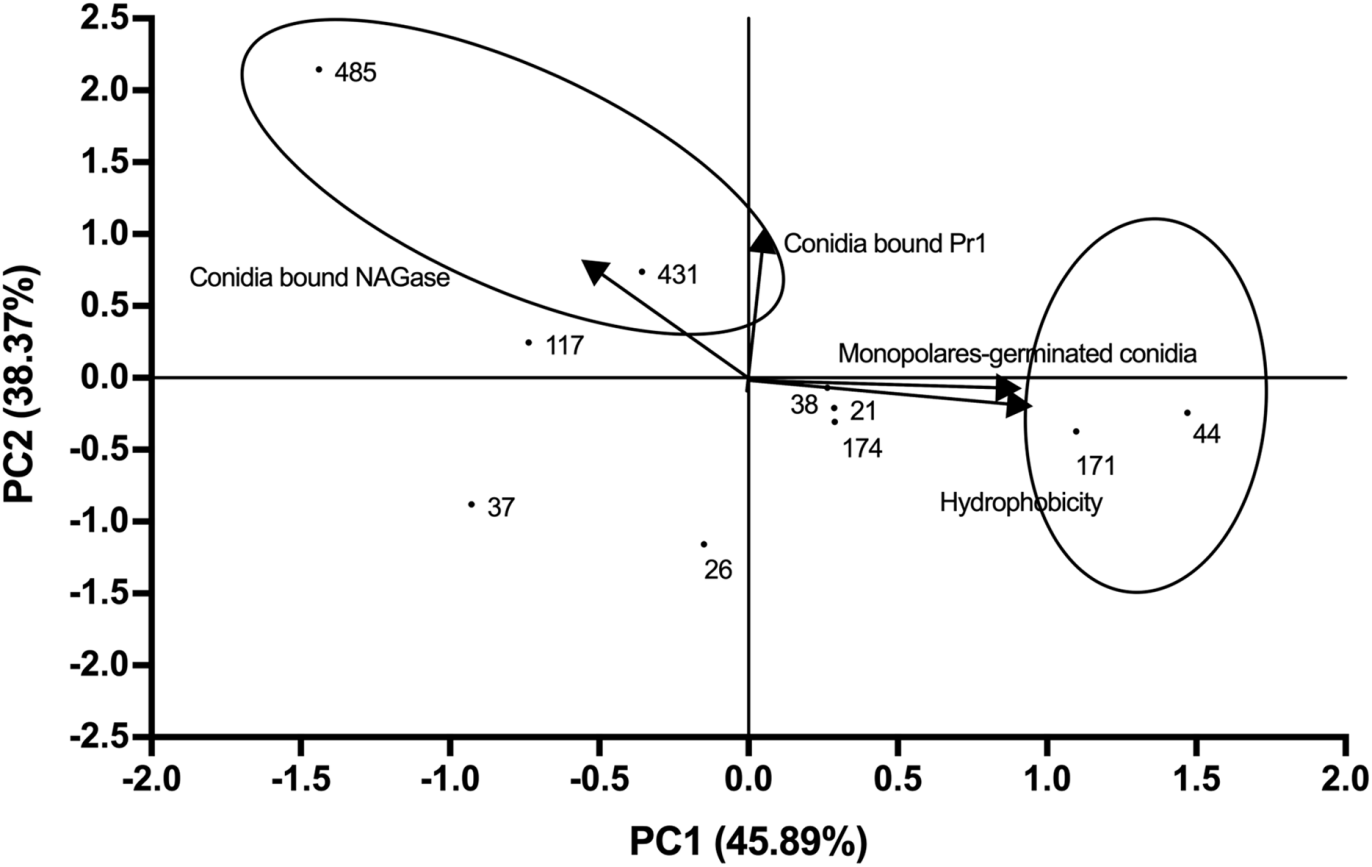
Relationships between the variables, components (PC1 vs. PC2) and distribution of *Beauveria bassiana* strains at the second selection stage. Each point represents the weighted sum of each strain in its respective component. The arrows indicate the direction and magnitude of the correlation of the variables with the extracted components.

The average values of the measured parameters Pr1-like proteases, NAGase chitinase, conidia hydrophobicity and monopolar conidial germination percentage for each of the 10 selected strains are shown in Table S4. PCA analysis revealed that 84.26% of the variability could be attributed to two main components (Fig. 3; Eigenvalue 0.96). A high correlation was seen between PC1 and the proportion of monopolar conidia as well as hydrophobicity (correlation coefficient of 0.90). For PC2, a correlation was seen with Pr1 protease activity and NAGase chitinase activity (correlation coefficient of 0.96) (Table S5).

Comparison of the distribution of the weighted sum of each strain for component showed that CHE-CNRCB 44 and 171 had higher hydrophobicity and monopolar conidial germination levels, whereas CHE-CNRCB 431 and 485 exhibited higher NAGase chitinase and Pr1 enzymatic activity, respectively (locked in the ovals of Fig. 3). The described strains distribution by the evaluated variables in this stage allowed their selection for the subsequent *in vivo* tests.

### Bioassays against *X. affinis*

*Xyleborus affinis* females were susceptible to *B. bassiana* infection when applied at 1 ${\rm \times}$ 10^8^ conidia mL^−1^, using spray application as the inoculation method. The average mortality rate (*n* = 100) among the evaluated *B. bassiana* strains ranged between 40% and 58% (Table 1). Strains 485, 171 and 431 caused mortality rates greater than 40%, with 58 ± 13.7%, 48 ± 9.0% and 48 ± 9.7% mortality rates, respectively, whereas strain 44 induced the lowest mortality (40 ± 13.3%). Mortality in the control was 19.6 ± 2.7%. In order to confirm that beetle mortality was by fungal infection, *B. bassiana* sporulation was analysed from dead washed insects incubated inside a humidified chamber. Growth of *B. bassiana* on dead beetles resulted in aerial mycelia development, ranging between 12% (CHE-CNRCB 44) to 30% (CHE-CNRCB 485).

**Table 1:** Virulence of four *Beauveria bassiana* strains against *Xyleborus affinis* females.

Strain	Parameters^a^					
	Mor (%)	Mic (%)	*k* (d ^−1^)	*t*_o_ (d)	LT_40_ (d)	Con/ins (×10^7^)
44	40 ± 13.3^b^	12 ± 10.9^b^	0.29 ± 0.04^a^	1.95 ± 0.6^a^	7.97 ± 2.5^a^	0.47 ± 0.3^a^
171	48 ± 9.0^bc^	15 ± 7.9^bc^	0.37 ± 0.15^a^	3.05 ± 1.1^ab^	9.62 ± 2.8^a^	1.62 ± 0.7^ab^
431	48 ± 9.7^bc^	25 ± 12.7^cd^	0.52 ± 0.03^a^	4.78 ± 0.2^b^	6.49 ± 2.1^a^	4.74 ± 0.8^c^
485	58.7 ± 13.7^c^	30 ± 4.1^d^	0.58 ± 0.01^a^	2.93 ± 0.07^ab^	5.32 ± 0.2^a^	0.82 ± 0.08^ab^
Control	19.6 ± 2.7^a^	0^a^	–	–	–	–

**Note:**

a Mor = mortality (percentage); Mic = *B. bassiana* mycosis (percentage); *k* = specificity death rate (day^−1^); *t*_0_ = dying delay time (days). Means ± SD within a column followed by the same upper letter were not statistically different (*p* < 0.05).

Lethal Time 40 (LT_40_) averages between 5.32 and 9.62 d were observed by strains CHE-CNRCB 485 and CHE-CNRCB 171, respectively. Strain 44 caused the fastest rate of mortality, where treated insects started dying from the second day (*t*_0_ = 1.95). Beetles treated with CHE-CNRCB 431 begin dying around the fifth day, following treatment. The amount of conidia counted *per* treated beetle demonstrated that strain 431 caused the highest conidia production *per* beetle corpse (4.74 ± 0.8 $\times$ 10^7^ conidia insect^−1^).

## Discussion

Numerous factors influence the infection process of insects by entomopathogenic fungi (EPF), and the measurement of these factors presents an opportunity to characterise these microorganisms and to identify promising mycoinsecticides (Peteira et al., 2011). Growth variables are EPF attributes that often indicate potential for their use (Wraight, Inglis & Goettel, 2007; Ravensberg, 2011). Among them, infective units’ production is an important attribute for EPF selection, since it influences the subsequent commercial-scale production (Kamp & Bidochka, 2002).

The first selection stage carried out in this work involved the specific growth profile required for a mycoinsecticide: good growth, sporulation and germination rates, which represent basic aspects for its massive production (Feng, Poprawski & Khachatourians, 1994; Montesinos, 2003). Initial PCA results allowed us to establish a positive correlation between only two of the four parameters evaluated, involving germ tube length and germination. Thus, some *B. bassiana* strains were categorised into three clusters according to the following features: (I) long germ tube length and high germination, (II) high growth rate, and/or (III) high conidial production. Some of the strains were found in more than one category, they were also considered good candidates, and will be evaluated against other species of ambrosial beetles (Montesinos-Matías et al., 2019b). Moreover, the most virulent strains are under scale-up production process including the use of cooked rice, and in plastic bags (Arredondo-Bernal & Rodríguez-Vélez, 2020). In other studies, Almeida, Alves & Pereira (1997) reported a positive correlation between *Beauveria* spp. conidial production and *Heterotermes tenuis* (Hagen) mortality. Pletamul & Prasertsan (2012) reported that the *B. bassiana* strain BNBCRC, which exhibited high viability and moderate radial growth, resulted in *S. litura* increased mortality. Here, we hypothesised that the 10 strains selected in the first PCA stage analysis, may be virulent against *X. affinis*. However, we selected other more closely related variables analysis during insects’ infectious processes.

The hydrolytic activity of the EPF enzymes has been considered a parameter related to potential insecticidal effect. In this regard, cuticle-degrading enzymes are a key factor of fungal infection to insects (St. Leger et al., 1991; Montesinos-Matías et al., 2011a), activity that may result in a time to kill reduction (Montesinos-Matías et al., 2011a; Castrejón-Antonio et al., 2017a). Previous reports of enzyme overexpression have demonstrated the role of cuticle-degrading enzymes (hydrolases such as Pr1 protease and chitinases) in the virulence of *B. bassiana* against target insects (Fang et al., 2009; Pelizza et al., 2012). Based on the second PCA results, a correlation was observed between the protease and NAGase chitinase production, and between polarity and hydrophobicity. The presence of hydrophobins on the cell wall of fungal conidia is involved in the adhesion to the insects’ cuticle, leading to a higher insect invasion probability (Butt et al., 2016; Wang & Wang, 2017). The relationship between virulence and germ tube polarity is possibly due to the metabolic activity in a single germ tube, which facilitates the penetration into the insect’s body (Butt et al., 2016; Wang, Lovett & St. Leger, 2019). Out of the four strains evaluated on the beetles, two were located in each of the two clusters and being active against *X. affinis* females. PCA analysis upon the mortality parameters results showed that the strain 485 had the highest enzymatic activity and resulted in the highest beetle mortality among the four selected strains. Strain 485, in addition to 431 (the second showing the highest enzymatic production) resulted in the highest mycosis percentage among treated insects. It seems that the enzymes related to cuticle degradation, are an adequate indicator of virulence as reported previously (Zare, Talaei-Hassanloui & Fotouhifar, 2014). It was suggested that during the selection of EPF for mycoinsecticides development, the hydrolases production profiles by the candidate strains are crucial, and it is important to also consider additional profiles involved in the infectious process, such as those of lipases, catalases or cytochromes (Ortíz-Urquiza & Keyhani, 2013; Keyhani, 2017). In addition, the selection strategy followed in this work had the advantage that the selected fungi may favourably grow in the subsequent evaluation stages. Other advantage of the selected strains is that most of them were molecularly identified (Serna-Domínguez et al., 2019) and a high diversity detected.

Since fungal genomes are markedly dynamic (Kary & Alizadeh, 2017), genetic modification has been achieved to enhance fungi virulence and tolerance to adverse conditions (St. Leger & Wang, 2010; Zhao, Lovett & Fang, 2016), and determine phenotypes selection for screening fungal isolates and their potential development as mycoinsecticides. In addition to the strains’ stability or virulence after their manipulation upon culture conditions, morphological and physiological variants are frequently observed after artificial media culture for maintenance purposes (e.g. Pr1 is produced under carbon and nitrogen de-repression, specifically induced by proteinaceous component of the insect cuticle).

During the comparison of insecticide activity levels by the entomopathogens, we must consider the infective unit’s application pathway, as well as the conditions that the host undergoes during the time the evaluation lasts. In this work, the direct spraying of infective units on the beetle was used, with the purpose of giving opportunity to the insects to generate galleries and produce their food during the time of the bioassay. The use of this protocol has the disadvantage of bringing small amounts of conidia into the insect, firstly because of a small contact surface and secondly, by the release of conidia that the insects leave by the contact with the diet to generate galleries. Under the conditions tested, insecticidal activity was achieved on *X. affinis*, with mortality results slightly lower than the reported by other researchers who used commercial *B. bassiana* strains in other species of ambrosia beetles. Carrillo et al. (2015) reported mortality rates between 54.7% and 70.7% for *X. glabratus* that had been exposed to the *B. bassiana* GHA (BotaniGard ES) strain, whereas Castrillo et al. (2011) and Carrillo, Crane & Peña (2013) reported 6–60% mortality rates for *Xylosandrus germanus* with *B. bassiana* GHA and 76.7–95.6% for *Xylosandrus crassiusculus*, using Naturalis^®^, respectively.

It is highlighted that the bioassay protocols of the aforementioned works were different, for example using inoculation by immersion (Carrillo et al., 2015) or aspersion (Castrillo et al., 2011; Carrillo, Crane & Peña, 2013), keeping the insects during the bioassay time only on wet filter paper pieces, without food, which stressed beetles and compromised their immune system (Adamo et al., 2016; Deans et al., 2017). Based on this information, we consider that the bioassay method on avocado’s sawdust artificial diet better simulates the interaction of the entomopathogen with the beetle and allows us to determine the effect on their cryptic ecology. Indeed, we believe that using indirect insect inoculation on surfaces contaminated with entomopathogens will provide even more information about the impact of these applications, simulating the conditions of a field application, for example on wood trunks, considering that this pest colonises most of its galleries.

Using the same bioassays and *B. bassiana* selected strains that we described in this study, against the ambrosia beetle *Euwallacea kuroshio* Gomez and Hulcra, Montesinos-Matías et al. (2019b) reported higher than 80% mortality. Following the bioassay from the *B. bassiana* treated females, offspring (eggs and larvae) population decreased more than 65%.

## Conclusions

The present work appears to be the first in Mexico to evaluate the potential of EPF for the control of native ambrosial beetles *X. affinis*, a beetle that affects active avocado orchards and potentially transports and transmits the *R. lauricola* fungus.

It was possible to discriminate strains of *B. bassiana* employing physiological and biochemical attributes as indicators of mass production and insecticidal activity, using PCA as exploratory statistical tool. Particularly, for bioassays with scolytinae beetles, due to their cryptic ecology, special care must be taken to provide information as helpful as possible to meet their potential effect in the field, recommending the use of strategies that do not limit the access to insects’ food and employed indirect inoculation, using surfaces with propagules of EPF.

As a perspective, one of the strategies to improve the efficacy of the selected *B. bassiana* strains on *X. affinis* is the use of formulations and their comparison with non-formulated infective units is projected, in addition to performance evaluation of our strains against other ambrosia beetle complexes.

## Supplemental Information

10.7717/peerj.9472/supp-1 Supplemental Information 1 Analysed variables in the first phase of *Beauveria bassiana* selection.

10.7717/peerj.9472/supp-2 Supplemental Information 2 Correlation coefficients between the analysed variables (initial stage of selection) and the extracted principal components 1, 2 and 3 (PC1, PC2, PC3) after Varimax rotation.

10.7717/peerj.9472/supp-3 Supplemental Information 3 Grouping of the *Beauveria bassiana* strains according to the highest measured variables..

10.7717/peerj.9472/supp-4 Supplemental Information 4 Measured variables in the second phase of *Beauveria bassiana* selectio.

10.7717/peerj.9472/supp-5 Supplemental Information 5 Correlation coefficients between the analysed variables (second selection stage) and the extracted principal components 1 and 2 (PC1, PC2) after Varimax rotation.

10.7717/peerj.9472/supp-6 Supplemental Information 6 Analyzed data. Principal components stages 1 and 2

## Additional Information and Declarations

### Field Study Permissions

The following information was supplied relating to field study approvals (i.e., approving body and any reference numbers): A permit to collected beetles was granted by the Colima State Plant Health Committee (Comité Estatal de Sanidad Vegetal de Colima) and the landowner, Julio Aguirre González.

### Data Availability

The following information was supplied regarding data availability: Raw data is available in the Supplemental Files.
