# Supplementary material for: Selection of Beauveria bassiana (Hypocreales: Cordycipitaceae) strains to control Xyleborus affinis (Curculionidae: Scolytinae) females
Source: PeerJ. 2020 Jul 3;8:e9472. doi: 10.7717/peerj.9472 (PMC7337030; doi:10.7717/peerj.9472)
Supplement: Supplemental Information 1 [file peerj-08-9472-s001.docx]

| **Table S1.** Analysed variables in the first phase of *Beauveria bassiana* selection. | | | | |
| --- | --- | --- | --- | --- |
|  | **Variables^a^** | | | |
| **Strain** | **Growth rate**  **(mm day ^-1^)** | **Conidial production**  **(con mm^-2^) (×10^8^)** | **Germination**  **(%)** | **Germ tube length (mm)** |
| 21 | 1.98±0.15^a^ | 11.7±1.23^def^ | 97.56 ±0.38^a^ | 55.28±11.25^f^ |
| 22 | 3.09±0.24^bcdef^ | 3.35±1.18^ab^ | 96.11±3.27^ab^ | 71.22±24.19^gh^ |
| 25 | 3.65±0.69^ef^ | 2.73±0.59^ab^ | 93.11±3.01^ab^ | 34.49±8.92^bcd^ |
| 26 | 2.25±0.31^ab^ | 7.07±1.06^bcd^ | 96.78±1.35^ab^ | 67.28±18.40^fghi^ |
| 27 | 2.18±0.14^ab^ | 3.00±1.29^ab^ | 94.83±3.55^ab^ | 66.65±23.96^fghi^ |
| 30 | 2.14±0.68^ab^ | 1.61±0.44^ab^ | 87.11±3.42^b^ | 30.71±8.35^abcd^ |
| 37 | 3.73±0.13^ef^ | 7.18±1.23^bcd^ | 96.39±1.78^ab^ | 76.96±19.56^gh^ |
| 38 | 3.02±0.40^abcdef^ | 5.56±0.78^abc^ | 96.00±2.00^ab^ | 57.82±19.15^fg^ |
| 44 | 2.47±0.30^abcd^ | 15.7±6.92^ef^ | 88.56±3.67^ab^ | 20.61±6.85^a^ |
| 105 | 3.32±0.47cd^ef^ | 2.62±0.99^ab^ | 94.11±0.96^ab^ | 69.43±18.98^fgh^ |
| 117 | 2.86±0.29^abcdef^ | 12.1±1.68^cde^ | 96.28±0.75^ab^ | 33.08±9.47^abcd^ |
| 171 | 3.36±0.03^cdef^ | 16.9±2.75^f^ | 92.94±4.03^ab^ | 25.33±4.73^ab^ |
| 173 | 2.70±0.29^abcde^ | 0.21±0.06^a^ | 95.44±2.22^ab^ | 27.08±5.95^abc^ |
| 174 | 3.44±0.21^def^ | 13.0±1.63^ef^ | 89.22±5.58^ab^ | 28.35±8.15^abcd^ |
| 431 | 3.77±0.18^f^ | 3.71±1.00^ab^ | 89.89±9.05^ab^ | 42.14±12.35^e^ |
| 483 | 2.72±0.20^abcde^ | 2.58±0.75^ab^ | 94.44±1.95^ab^ | 39.68±8.47^cd^ |
| 484 | 3.05±0.44^bcdef^ | 3.38±2.6^ab^ | 95.33±1.33^ab^ | 40.87±10.66^d^ |
| 485 | 3.11±0.24^bcdef^ | 1.42±0.12^ab^ | 96.56±0.51^ab^ | 78.96±16.62^h^ |
| 486 | 2.32±0.60^abc^ | 2.70±0.02^ab^ | 97.00±0.67^ab^ | 64.64±12.75^fgh^ |

^a^Means ± SD within a column followed by the same upper letter were not statistically different (*p*<0.05).
