# Supplementary material for: Selection of Beauveria bassiana (Hypocreales: Cordycipitaceae) strains to control Xyleborus affinis (Curculionidae: Scolytinae) females
Source: PeerJ. 2020 Jul 3;8:e9472. doi: 10.7717/peerj.9472 (PMC7337030; doi:10.7717/peerj.9472)
Supplement: Supplemental Information 2 [file peerj-08-9472-s002.docx]

| **Table S2.** Correlation coefficients between the analysed variables (initial stage of selection) and the extracted principal components 1, 2 and 3 (PC1, PC2, PC3) after Varimax rotation. | | | |
| --- | --- | --- | --- |
| **Variable** | **PC1** | **PC2** | **PC3** |
| **Growth rate** | -0.04 | 0.98 | 0.09 |
| **Conidial production** | -0.09 | 0.09 | 0.98 |
| **Germination** | 0.91 | -0.29 | 0.08 |
| **Length of germ tube** | 0.89 | 0.21 | -0.27 |
