# Supplementary material for: Selection of Beauveria bassiana (Hypocreales: Cordycipitaceae) strains to control Xyleborus affinis (Curculionidae: Scolytinae) females
Source: PeerJ. 2020 Jul 3;8:e9472. doi: 10.7717/peerj.9472 (PMC7337030; doi:10.7717/peerj.9472)
Supplement: Supplemental Information 3 [file peerj-08-9472-s003.docx]

| **Table S3*.*** Grouping of the *Beauveria bassiana* strains according to the highest measured variables*.* | | |
| --- | --- | --- |
| **Germination (%) and length of germ tube (mm)** | **Growth rate**  **(mm day ^-1^)** | **Conidial production**  **(con mm^-2^) (×10^8^)** |
| 37 | 431 | 171 |
| 485 | 37 | 117 |
| 26 | 25 | 44 |
| 21 | 105 | 21 |
| 486 | 174 | 174 |
| 22 | 485 | 26 |
| 38 | 171 | 38 |
