# Supplementary material for: Selection of Beauveria bassiana (Hypocreales: Cordycipitaceae) strains to control Xyleborus affinis (Curculionidae: Scolytinae) females
Source: PeerJ. 2020 Jul 3;8:e9472. doi: 10.7717/peerj.9472 (PMC7337030; doi:10.7717/peerj.9472)
Supplement: Supplemental Information 4 [file peerj-08-9472-s004.docx]

| **Table S4.** Measured variables in the second phase of *Beauveria bassiana* selection. | | | | | |
| --- | --- | --- | --- | --- | --- |
| **Strain** | **Variables^a^** | | | | |
|  | **Conidia bound Pr1**  **(nmoles NA mL^-1^**  **min^-1^)** | **Conidia bound NAGases**  **(nmoles NP mL^-1^ min^-1^)** | **Relative hydrophobicity**  **(%)** | | **Unipolar-germinated**  **Conidia (%)** |
| 21 | 107.89±3.16^c^ | 9.99±0.43^a^ | 88.17±2.4^abc^ | 76.13±11.93^abc^ | |
| 26 | 74.67±2.74^a^ | 7.16±1.26^a^ | 87.50±1.75^abc^ | 60.13±9.82^abc^ | |
| 37 | 81.64±2.91^ab^ | 9.97±1.04^a^ | 76.33±9.52^bc^ | 56.88±10.67^bc^ | |
| 38 | 112.64±1.11^c^ | 11.21±2.39^abc^ | 90.28±9.09^ab^ | 66.63±14.27^abc^ | |
| 44 | 107.67±6.14^c^ | 7.71±1.84^a^ | 96.23±4.86^a^ | 85.50±6.59^a^ | |
| 117 | 110.00±2.1^c^ | 16.15±1.01^c^ | 67.42±6.79^c^ | 82.17±2.71^c^ | |
| 171 | 110.89±2.92^c^ | 6.63±1.64^a^ | 92.25±7.75^ab^ | 84.50±6.14^ab^ | |
| 174 | 90.94±9.40^b^ | 11.10±2.64^ab^ | 86.67±0.76^abc^ | 83.50±4.69^abc^ | |
| 431 | 111.64±4.73^c^ | 19.78±0.53^d^ | 86.17±2.88^abc^ | 65.63±6.61^abc^ | |
| 485 | 245.25±4.79^d^ | 17.68±2.22^d^ | 74.42±3.41^bc^ | 7.13±2.47^d^ | |

^a^Means ± SD within a column followed by the same upper letter were not statistically different (*p*<0.05). NA: Nitroaniline, NP: Nitrophenol
