# Supplementary material for: Selection of Beauveria bassiana (Hypocreales: Cordycipitaceae) strains to control Xyleborus affinis (Curculionidae: Scolytinae) females
Source: PeerJ. 2020 Jul 3;8:e9472. doi: 10.7717/peerj.9472 (PMC7337030; doi:10.7717/peerj.9472)
Supplement: Supplemental Information 5 [file peerj-08-9472-s005.docx]

| **Table S5.** Correlation coefficients between the analysed variables (second selection stage) and the extracted principal components 1 and 2 (PC1, PC2) after Varimax rotation. | | |
| --- | --- | --- |
| **Variable** | **PC1** | **PC2** |
| **Conidia bound Pr1** | 0.04 | 0.96 |
| **Conidia bound NAGases** | -0.53 | 0.77 |
| **Hydrophobicity** | 0.90 | -0.17 |
| **Unipolar-germinated conidia** | 0.85 | -0.04 |
